# The effect of the kindergarten barefoot policy on preschool children’s toes

**DOI:** 10.1186/s40101-016-0097-3

**Published:** 2016-07-07

**Authors:** Shigeki Matsuda, Kosho Kasuga, Tadayuki Hanai, Tomohiro Demura, Keisuke Komura

**Affiliations:** Shiga University, 2-5-1, Hiratsu, Otsu, Shiga 520-0862 Japan; Gifu University, 1-1, Yanagido, Gifu, Gifu 501-1193 Japan; Chubu University, 1200, Matsumoto-cho, Kasugai, Aichi 487-8501 Japan; Jin-ai University, 3-1-1, Ohde-cho, Echizen-city, Fukui 915-8586 Japan; Kyoto Bunkyo Junior College, 80, Senzoku, Makishima-Cho, Uji-City, Kyoto 611-0041 Japan

**Keywords:** Foot shape, Barefoot, Toe, Untouched-toe, Infant

## Abstract

**Background:**

This study compared the effects of barefoot policy, a policy instructing preschool children to go without shoes, on untouched-toes, which do not touch the ground while standing normally, of preschool children attending kindergartens that follow this rule, to preschooler in kindergartens where they must wear shoes, i.e., no-barefoot policy.

**Methods:**

The study used longitudinal data from measurements taken 2 years apart of the amount of times. The subjects were 59 children (34 boys and 25 girls) who went to a kindergarten that followed barefoot policy and 179 children (103 boys and 76 girls) who went to a kindergarten that did not follow barefoot policy. Images were taken of the contact surface area of the soles of the children’s feet by having them stand on the measurement device with their bare feet.

**Results:**

The number of untouched-toes in children participating in the study was determined from the pictures. In boys who attended kindergartens following barefoot policy, the ratio of the children without untouched-toes significantly increased for 2 years of childhood (35.3–64.7 %). The number of untouched-toes were significantly fewer in boys from kindergartens following barefoot policy than in boys from kindergartens not following the policy, and the magnitude of the difference grew for the two study years (ES: 0.41–0.63). In girls, there were no significant differences between the two groups in the ratio of girls without untouched-toes and the number of untouched-toes.

**Conclusions:**

In conclusion, the ground contact of the toes becomes better for boys in kindergarten with a barefoot policy. The results were inconclusive with regard to girls, and other factors may need to be examined. In the future, it will be necessary to increase the number of the subjects and perform detailed examinations.

## Background

The term “untouched-toes” refers to when the toes do not touch the ground while standing normally [1–3]. Also, they do not seem to cause pain or need surgery. Untouched-toes are also called “floating-toes” [4]. In Japan, the number of children with untouched-toes is on the rise [2, 5, 6]. Some factors related to this condition have been pointed out, such as shoes, heel load, the amount of activity, posture, etc. [7]. Among these factors, the relationship between the untouched-toes and the heel load has been examined, and it is reported that children with over two untouched-toes experience more heel load than children without any untouched-toes [3]. There is an expectation that the association of heel load with untouched-toes originates from inadequate activity of muscles and bad posture. In addition, this condition is undesirable because foot pressure load moves anteriorly with aging in childhood [8, 9]. In a previous study on young women, Akamatsu and Nakatsuka [1] reported a relationship between untouched-toes and general malaise. Lower back pain caused due to untouched-toes has been known to be relieved after wearing sandals and exercising the toes [10]. These studies suggest that untouched-toes have negative health effects.

There have always existed kindergartens that follow the barefoot policy in Japan. Although it is reported that going barefoot affects the formation of the medial longitudinal arch [11], the effect of barefoot policy for kindergartens has not been sufficiently or multilaterally examined. The relationship between barefoot policies and untouched-toes has also not been thoroughly examined. Generally speaking, people can move their toes more freely and frequently when they are barefoot compared to when they wear shoes. The decrease in frequency of use of the toes caused by the decrease in general levels of activity or the effect of wearing shoes have been discussed as causes for untouched-toes [7]. Hence, there is a possibility that the occurrence of untouched-toes can be decreased by increasing the barefoot activity of children. In addition, because they are still growing, the shape of children’s feet is easily changed by external stimuli as the growth of bones is marked in childhood, and their bones contain a substantial amount of cartilage. It is not hard to deduce that the quantity of barefoot activity is likely to decrease the occurrence of untouched-toes. This study clarified the effect of kindergarten barefoot policy on the untouched-toes of preschool children by comparing children who attend kindergarten following the barefoot policy and those attending kindergarten that follow the no-barefoot policy.

## Methods

### Subjects

The subjects were 59 children (34 boys and 25 girls) in a kindergarten that followed barefoot policy (barefoot group) and 179 children (103 boys and 76 girls) in a kindergarten that did not follow barefoot policy (non-barefoot group). The subjects in the barefoot group played barefoot indoors after arriving at their kindergarten. They did not even wear their socks. According to the interviews of the kindergarten’s staff, not many severe accidents and injuries were caused by the barefoot policy. The subjects in the non-barefoot group played in shoes. The kind of shoes (sports shoes, sandals, etc.) worn by each child was not particularly designated. Generally, children spend 5 h or more in their kindergarten on weekdays. Whether a child in either group spends time at home barefoot or wearing shoes (socks) is completely dependent on the child. Table 1 shows the number of subjects and their physical characteristics. Age in months was used to provide the age of each subject. In this study, the subjects were 4 years old at the first measurement and were measured again 2 years later. There were significant differences between the two groups in the body mass of the subjects, i.e., the boys and girls, in the first measurement. Moreover, the height for boys and the body mass for girls in the second measurement also exhibited significant differences. However, as physique has not been reported to affect the occurrence ratio of untouched-toes [2, 5], the difference in physique of the subjects was not considered in the following analysis. The purpose and procedure of this study were explained to the children’s parents and kindergarten teachers in detail and informed consents were obtained before the study began or any measurements were taken. This experimental protocol was approved by the Ethics Committee on Human Experimentation of Faculty of Human Science, Kanazawa University (2012-5).

**Table 1:** Number of subjects and their physical characteristics

			Barefoot group		Non-barefoot group	
			1st 2 years later		1st 2 years later	
Boys	n		34	34	103	103
	Age	Mean	4.2	6.2	4.4	6.4
		S.D.	0.3	0.3	0.2	0.2
	Height	Mean	100.8	113.8	102.2	115.7
	(cm)	S.D.	4.1	4.6	4.0	4.6
	Body mass	Mean	15.9	20.3	17.0	21.3
	(kg)	S.D.	2.1	3.8	1.9	3.0
Girls	n		25	25	76	76
	Age	Mean	4.3	6.3	4.4	6.4
		S.D.	0.2	0.2	0.1	0.1
	Height	Mean	101.0	114.5	102.5	116.3
	(cm)	S.D.	4.0	4.3	3.7	4.6
	Body mass	Mean	15.7	19.6	17.0	21.7
	(kg)	S.D.	1.9	2.4	1.8	2.7

### The measurement of contact surface area of the sole

A pedoscope (Sakamoto, Japan) was used to record the contact surface area of the soles of the subjects’ feet, which was used to analyze the number of untouched-toes. The subjects stood barefoot on the device with their feet 5 cm apart and their hands relaxed at their sides. They were instructed to look at a mark located at eye-level and to remain as still as possible during the measurement. After a tester, who was a university teacher, confirmed the subject’s postural stability, a picture of the contact surface area of the soles of their feet was recorded. Each subject was sequentially measured five times while standing.

### Judgment of untouched-toes

Untouched-toes refer to the toes that do not show up in more than four of the five recorded pictures. A subject was defined as having untouched-toes when there was more than one untouched-toe among the toes of both feet. The sum of the untouched-toes on both feet was evaluated as the total number of untouched-toes.

### Statistical analysis

Fisher’s exact test was used to examine the difference between the first measurements and the measurements 2 years later for the ratio of subjects with/without untouched-toes. A two-way analysis of variance was used to examine the differences between the two groups and between the first and second measurements of the number of untouched-toes. If a significant difference was found, the size of the effect was calculated to examine the size of the mean difference. Independent testing was used to examine the differences between the two groups in the ratio of the number of untouched-toes (0 untouched-toe, one untouched-toe, and over two untouched-toes). A test of goodness of fit was used to examine the differences of the ratio between three categories (“increased,” “no change,” and “decreased”) classified according to changes in the number of untouched-toes between the first and second measurements. If a significant difference was found, multiple comparison tests were conducted. The level of statistical significance was set at *p* < 0.05.

## Results

Table 2 shows the results of the difference between the first and second measurements in the ratio of the children with/without untouched-toes in the barefoot group. There was a significant difference between the two measurement periods in the ratio of the children with/without untouched-toes in boys. The number of children without untouched-toes changed from 12 (35.3 %) in the first measurement to 22 (64.7 %) when measured 2 years later. In girls, although the number of children without untouched-toes increased from seven (28.0 %) in the first measurement to 11 (44.0 %) in the second measurement, a significant difference was not found.

**Table 2:** Results of the difference between the first measurement and the measurement two years later in the ratio of the children with/without untouched-toes in barefoot group

Boys						Girls					
		2 years later						2 years later			
		without U-toes	with U-toes	sum	p			without U-toes	with U-toes	sum	p
1st	without U-toes	11	1	12 (35.3%)	0.01*	1st	without U-toes	6	1	7 (28.0%)	0.22
	with U-toes	11	11	22 (64.7%)			with U-toes	5	13	18 (72.0%)	
	sum	22 (64.7%)	12 (35.3%)	34			sum	11 (44.0%)	14 (56.0%)	25	

Note) U-toes: Untouched-toes, *p< 0.05

Table 3 shows the results of the difference between the first and second measurements in the ratio of children with/without untouched-toes in the non-barefoot group. The number of children without untouched-toes was 24 (23.3 %) for boys and 27 (36.5 %) for girls in the first measurement and 28 (27.2 %) for boys and 29 (38.2 %) for girls 2 years later. There were no significant differences between the two measurements in the ratio of children with/without untouched-toes in the non-barefoot group.

**Table 3:** Results of tire difference between tire first measurement and tire measurement two years later n the ratio of the children with/without untouched-toes n non-barefoot group

Boys						Girls					
		2 years later						2 years later			
		without U-toes	with U-toes	sum	p			without U-toes	with U-toes	sum	p
1st	without U-toes	14	10	24 (23.3%)	0.54	1st	without U-toes	17	10	27 (35.5%)	0.83
	with U-toes	14	65	79 (76.7%)			with U-toes	12	37	49 (64.5%)	
	sum	28 (27.2%)	75 (72.8%)	103			sum	29 (38.2%)	47 (61.8%)	76	

Note) U-toes: Untouched-toes, *p< 0.05

Table 4 shows the results of the differences between the two groups and between the two measurement periods in the number of untouched-toes. In boys, the numbers were significantly fewer 2 years later than in the first measurement and in the barefoot group compared to the non-barefoot group. Although the effect size of the difference between the two groups in the first measurement was 0.41, it became 0.63 in the second measurement. In girls, there were no significant differences between the two groups or the two measurement periods in the number of untouched-toes. The results of the change in the number of untouched-toes are summarized in Table 5. Table 6 shows the results of the differences between the two groups in the ratio of the number of the untouched-toes (0 untouched-toe, one untouched-toe, and over two untouched-toes). A significant difference between the two groups for boys was found in the ratio of the number of untouched-toes. The ratio of over two untouched-toes was less in the barefoot group (six boys (17.6 %)) than in the non-barefoot group (47 boys (45.6 %)).

**Table 4:** Results of the differences between two groups and between two measurement perods n the number of the untouched-toes

		Barefoot group		Non-barefoot group			Two-way ANOVA		
		1st	2 years later	1st	2 years later		Group	Period	Interaction
Boys	Mean	1.35	0.68	2.04	1.42	F	8.03	24.05	0.04
	S.D.	1.57	1.09	1.69	1.19	p	0.01*	0.00*	0.84
Girls	Mean	1.16	1.04	1.41	1.09	F	0.40	1.83	0.37
	S.D.	1.07	1.10	1.43	1.13	p	0.53	0.18	0.54

Note: *p< 0.05

**Table 5:** Results of the change of the number of the untouched-toes

		Barefoot group				Non-barefoot group			
		1st		2 years later		1st		2 years later	
		n	%	n	%	n	%	n	%
Boys	0 U-toe	12	35.3	22	64.7	24	23.3	28	27.2
	1 U-toe	11	32.4	6	17.6	21	20.4	28	27.2
	2 U-toes	6	17.6	1	2.9	20	19.4	30	29.1
	3 U-toes	0	0.0	5	14.7	15	14.6	11	10.7
	4 U-toes	3	8.8	0	0.0	15	14.6	5	4.9
	5 U -toes	1	2.9	0	0.0	4	3.9	1	1.0
	6 U-toes	1	2.9	0	0.0	4	3.9	0	0.0
	7 U-toes	0	0.0	0	0.0	0	0.0	0	0.0
Girls	0 U-toe	7	28.0	11	44.0	27	35.5	29	38.2
	1 U-toe	11	44.0	5	20.0	17	22.4	23	30.3
	2 U-toes	4	16.0	6	24.0	15	19.7	16	21.1
	3 U-toes	2	8.0	3	12.0	11	14.5	4	5.3
	4 U-toes	1	4.0	0	0.0	5	6.6	4	5.3
	5 U-toes	0	0.0	0	0.0	0	0.0	0	0.0
	6 U-toes	0	0.0	0	0.0	0	0.0	0	0.0
	7 U-toes	0	0.0	0	0.0	1	1.3	0	0.0

Note) U-toes: Untouched-toes

**Table 6:** Results of the differences between the two groups b the ratb of number of the untouched-toes

		1st					2 years later				
		0 U-toe	1 U-toe	Over 2 U-toes	*X* ^2^	*p*	0 U-toe	1 U-toe	Over 2 U-toes	*X* ^2^	*p*
Boys	Barefoot group	12	11	11	5.88	0.05	22	6	6	15.97	0.00*
		35.3	32.4	32.4			64.7	17.6	17.6		
	Non-barefoot group	24	21	58			28	28	47		
		23.3	20.4	56.3			27.2	27.2	45.6		
Girls	Barefoot group	7	11	7	4.46	0.11	11	5	9	0.99	0.61
		28.0	44.0	28.0			44.0	20.0	36.0		
	Non-barefoot group	27	17	32			29	23	24		
		35.5	22.4	42.1			38.2	30.3	31.6		

Note) U-toes: Untouched-toes, *p<0.05

The children were divided into three categories: “increased,” “no change,” and “decreased” in terms of the change in the number of untouched-toes. Table 7 shows the results of the test for goodness of fit and multiple comparison tests in the ratio of each category in each group. A significant difference was found in boys in the barefoot group and the categories “decreased” (50.0 %) and “no change” (41.2 %) were larger than “increased” (19.4 %). Significant differences were found in boys and girls in the non-barefoot group. “Decreased” (48.5 %) was more than “increased” in boys and “no change” (47.4 %) was more than “increased” in girls.

**Table 7:** Results of test of goodness of fit and multbk com parison tests i the rati of each category

		Barefoot group		Test of goodness of fit		Multiple comparison tests	Non-barefoot group		Test of goodness of fit		Multiple comparison tests	
		n	%				n	%				
Boys	Decrease	17	50.0	*X* ^2^	9.59	Decrease, No change > Increase	50	48.5	*X* ^2^	13.18	Decrease > Increase	
	No change	14	41.2	*p*	0.01*		33	32.0	*p*	0.00*		
	Increase	3	8.8				20	19.4				
Girls	Decrease	6	24.0	*X* ^2^	3.92		24	31.6	*X* ^2^	8.00	No Change > Increase	
	No change	13	52.0	*p*	0.14		36	47.4	*p*	0.02*		
	Increase	6	24.0				16	21.1				

Note) *p< 0.05

## Discussion

First, the discussion will focus on the results for the boys because most of the results differed between boys and girls. The ratio of children without untouched-toes was 35.3 % at the first measurement and 64.7 % 2 years later. The ratio significantly increased in the barefoot group after 2 years. On the other hand, the ratio for the non-barefoot group was 23.3 % at the first measurement and 27.2 % after 2 years. The change in the ratio for 2 years was very low and a significant difference was not found in the ratio for non-barefoot group. These differences can also be seen in Table 5. The number of children without untouched-toes in the barefoot group was slightly more even in the first measurement because about 1 year had passed after children started commuting to the kindergarten at the time of the first measurement. There is a possibility that the ratio was slightly more because they had already spent about a year in the kindergarten that followed the barefoot policy. Moreover, it is inferred that the number of children without untouched-toes increased after spending two more years of following the barefoot policy in kindergarten.

The number of untouched-toes in boys was significantly less in the barefoot group than in the non-barefoot group. Also, the effect size of the difference expanded from 0.41 to 0.63 for 2 years. In addition, unlike the tendency of the non-barefoot group, the ratios of “decreased” and “no change” were more than the ratio of “increased” in terms of the change in the number of untouched-toes for 2 years. From the above results regarding the ratios of children without untouched-toes and the number of untouched-toes, the condition of the children with untouched-toes who attended the kindergarten that followed the barefoot policy improved compared to the children who were not allowed to go barefoot in kindergarten. This factor is important because it affects the gait and pressure on the toes while running and walking, which differs when one is barefoot and when wearing shoes. There are differences in terms of kinematics and kinetics [12]. Barefoot runners experience increased ankle plantarflexion and frequently land using their forefoot at initial contact; in contrast, runners wearing shoes frequently land on their heels [13]. There are significant differences in the walking gait, step length, cadence, and peak planter pressure between being barefoot and wearing shoes [14]. Peak impulses at the heel and metatarsals are lower, and the chances of the forefoot spreading under load are larger when barefoot than when in shoes [15]. Because about 40 % of body weight is concentrated on the toes in the final stage of forefoot contact when standing [16], it is the toes that actually support the body and make good contact with the ground when walking barefoot. Hence, it is possible that the children who attended the kindergarten that allowed them to go barefoot land on their forefoot as the initial contact, and frequently use their toes when walking. The shape of the foot changes by continuing the above gait differences when walking and running. Forefoot width becomes wider in habitual barefoot walkers compared to habitual walkers who wear shoes [14, 17]. In addition, the development of the medial longitudinal arch differs depending upon whether one habitually spends time barefoot or not [18]. The point is that the more the toes touch the ground, more are the changes observed in them. It is especially important to remember that foot shape in children changes markedly in early childhood. Foot length increases linearly between the ages of 3 and 15, and the medial longitudinal arch develops rapidly in childhood [18–20]. Children’s bones contain a high degree of cartilage making them more pliable and easier to be changed by external pressure. Because shoes restrict the natural motion of the foot or forefoot and increases the pressure on the toes [14], there is a possibility that shoes trigger an increase in the number of untouched-toes. By understanding that the shape of the toes changes and the number of untouched-toes decreases in children who play barefoot in a kindergarten that follows barefoot policy, one might conclude that such a policy is important for the current and future health of the next generations.

Matsuda, Demura [3] reported that children with over two untouched-toes tend to have greater heel loads than children without untouched-toes. By referring to the previous studies [3], the difference between the two groups in the ratio of children with over two untouched-toes was analyzed to clarify which children experienced greater heel load. Table 6 shows the results. A significant difference between the groups was found in the ratio of the number of untouched-toes in each category (0 untouched-toe, one untouched-toe, and over two untouched-toes) when measured 2 years later. The ratio of over two untouched-toes was less in the barefoot group than in the non-barefoot group. Hence, children who have greater heel loads may be fewer when they can go barefoot. As the child gets older, the foot is pressurized anteriorly [8, 9], even commuting children to a kindergarten that follows barefoot policy is better for healthy child development.

There is evidence that untouched-toes can have negative effects on the rest of the body well after childhood. Akamatsu and Nakatsuka [1] examined the relationship between untouched-toes and general malaise in young women. Their results indicated that having untouched-toes related to general malaise, headache, low back pain, and shoulder stiffness. In addition, Yahagi, Nemoto [10] examined the connection between having untouched-toes and low back pain in men and women. They found that low back pain was relieved by wearing sandals and undergoing exercise therapy to reduce the number of untouched-toes. The frequency of the fifth toe touching the ground is more in athletes compared with non-athletes [21], which implies a possible relationship between untouched-toes and athletic ability. Although more detailed studies will be needed in the future, there is a possibility that increasing barefoot policies in schools may have a favorable influence on the body and overall health from childhood.

It was interesting that the results for the girls differed from the boys and that there were no significant differences between the two measurement periods in the ratio of the girls with/without untouched-toes in the barefoot group and the non-barefoot group; there were no significant differences observed between the two groups after 2 years in the number of untouched-toes. It seems that the barefoot policy had hardly any effect on the number of toes touching the ground in the girls. Although it is difficult to interpret these results, one factor may be that in kindergarten the girls were less active than boys [22] as culturally, girls generally prefer static play. Because girls do not have many opportunities for using their toes and do not use their toes strongly, the barefoot policy seems to not have benefitted them as much. However, the amount and content of physical activity were not measured in this study. In the future, analysis of physical activity will be necessary. In addition, there is a possibility that statistical significant differences were not found because the number of girls in the study (25) was less than the number of boys. Girls without untouched-toes increased from 28.5 % at the first measurement to 44.0 % 2 years later. If the number of girls increases in the future, significant differences may be found. In addition, the number of the untouched-toes in girls was 1.16, which was less even at the first measurement because the first measurement was conducted about a year after the children had entered the kindergarten. If the first measurement is conducted soon after entering the kindergarten, different results may be obtained. This is also a point to be examined in the future along with increasing the number of boys and girls in the barefoot group for more accurate statistical meaning.

In this study, it depends on the child as to whether a child spends time at home barefoot, wearing just socks, or wearing shoes with or without socks. In Japan, some children go barefoot and others wear socks at home, which may also have some influence on the results of this study. In the future, the effect of the kindergarten barefoot policy should include this information as part of the control condition for the study. Moreover, in the future, it is possible that knowing the number of untouched-toes may become an index, which shows the condition of a child’s growth and health based on the continuation of the studies on this topic.

## Conclusions

This study examined the effects of attending a kindergarten that follows barefoot policy, i.e., allowing children to go barefoot in school, on the syndrome of untouched-toes in childhood using longitudinal data from two sets of measurements taken 2 years apart. Findings showed that the ratio of children (boys in particular) without untouched-toes increased in children attending a kindergarten that followed a barefoot policy for 2 years of childhood; whereas children in kindergarten that did not follow such a policy did not have the same results. The number of untouched-toes was less in children (boys in particular) who went to kindergarten where they could go barefoot than in children who had to wear shoes. Moreover, the magnitude of the difference expanded when the children were measured 2 years later compared to the initial measurement. One conclusion based on the evidence in this study is that barefoot policies in kindergarten cause more toes to touch the ground, especially in boys. Follow-up studies with a larger demographic of children are recommended for the future.
